# T cell Allorecognition Pathways in Solid Organ Transplantation

**DOI:** 10.3389/fimmu.2018.02548

**Published:** 2018-11-05

**Authors:** Jacqueline H. Y. Siu, Veena Surendrakumar, James A. Richards, Gavin J. Pettigrew

**Affiliations:** Department of Surgery, University of Cambridge, Cambridge, United Kingdom

**Keywords:** T cell allorecognition, transplantation, indirect presentation, cytotoxic CD8 T cells, T follicular helper cell, germinal center, exhaustion, chronic allograft vasculopathy

## Abstract

Transplantation is unusual in that T cells can recognize alloantigen by at least two distinct pathways: as intact MHC alloantigen on the surface of donor cells via the direct pathway; and as self-restricted processed alloantigen via the indirect pathway. Direct pathway responses are viewed as strong but short-lived and hence responsible for acute rejection, whereas indirect pathway responses are typically thought to be much longer lasting and mediate the progression of chronic rejection. However, this is based on surprisingly scant experimental evidence, and the recent demonstration that MHC alloantigen can be re-presented intact on recipient dendritic cells—the semi-direct pathway—suggests that the conventional view may be an oversimplification. We review recent advances in our understanding of how the different T cell allorecognition pathways are triggered, consider how this generates effector alloantibody and cytotoxic CD8 T cell alloresponses and assess how these responses contribute to early and late allograft rejection. We further discuss how this knowledge may inform development of cellular and pharmacological therapies that aim to improve transplant outcomes, with focus on the use of induced regulatory T cells with indirect allospecificity and on the development of immunometabolic strategies.

**KEY POINTS**

- Acute allograft rejection is likely mediated by indirect and direct pathway CD4 T cell alloresponses.
- Chronic allograft rejection is largely mediated by indirect pathway CD4 T cell responses. Direct pathway recognition of cross-dressed endothelial derived MHC class II alloantigen may also contribute to chronic rejection, but the extent of this contribution is unknown.
- Late indirect pathway CD4 T cell responses will be composed of heterogeneous populations of allopeptide specific T helper cell subsets that recognize different alloantigens and are at various stages of effector and memory differentiation.
- Knowledge of the precise indirect pathway CD4 T cell responses active at late time points in a particular individual will likely inform the development of alloantigen-specific cellular therapies and will guide immunometabolic modulation.

## Introduction

Although innate recognition of alloantigen can exhibit properties more typically associated with adaptive immunity (1–3), the conventional T cell response to alloantigen is still considered critical for determining short and long-term outcomes for solid organ transplants. Transplantation is unusual in that alloantigen can uniquely be recognized by at least two pathways; the indirect and the direct. The oft-repeated mantra that the short-lived direct pathway is responsible for acute rejection and the longer lasting indirect pathway mediates chronic rejection is based upon little evidence and, as highlighted by the recent description of a third semi-direct pathway, is an oversimplification. Here we review recent advances in our understanding of how different T cell allorecognition pathways may contribute to rejection and consider how this knowledge may inform development of cellular and pharmacological therapies that aim to improve transplant outcomes.

## Pathways of T cell allorecognition

### Direct pathway

**Figure 1:**
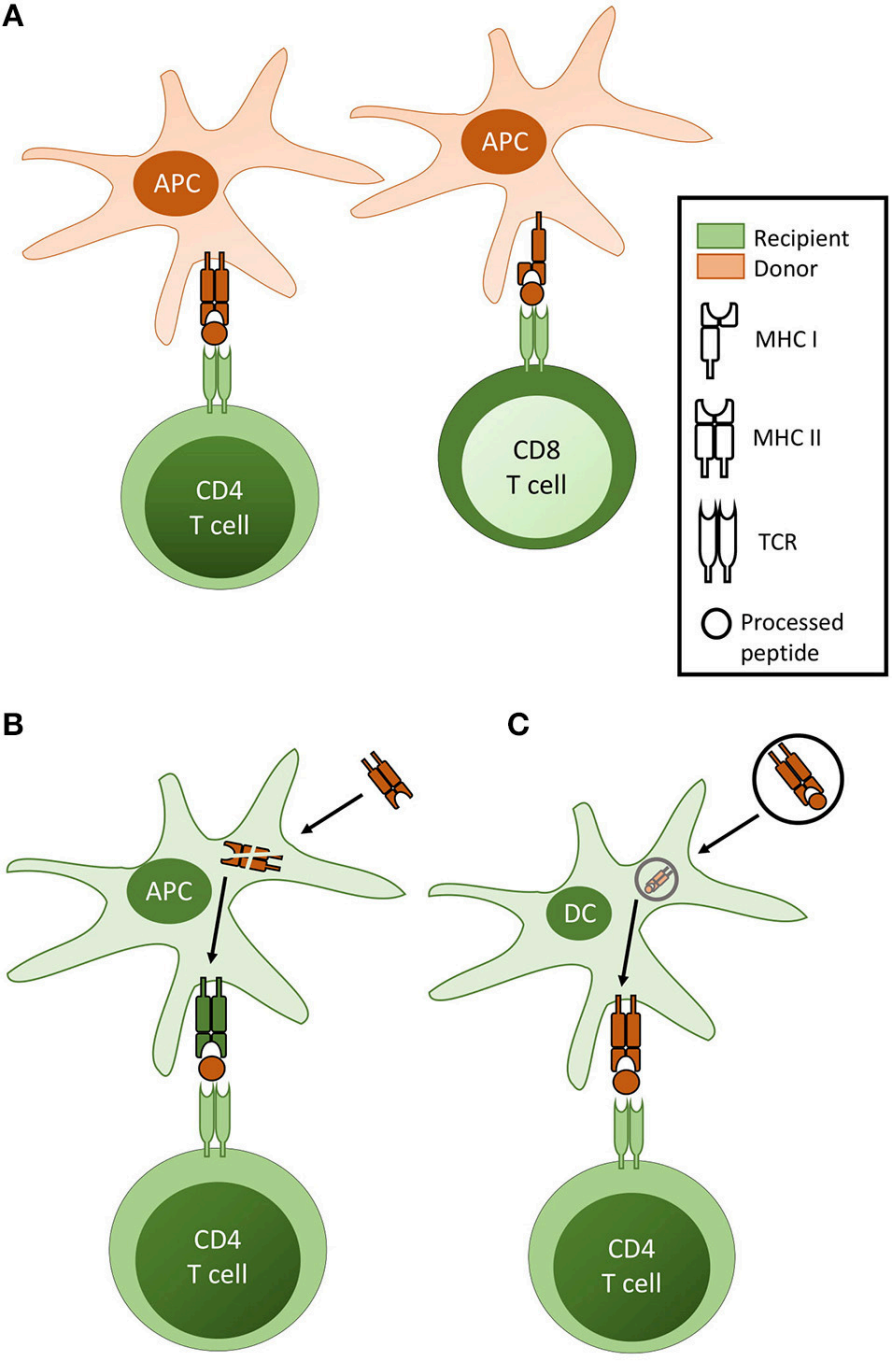
Pathways of T cell allorecognition. **(A)** In direct pathway allorecognition, MHC Class II and Class I alloantigen is recognised as intact protein on the surface of donor antigen presenting cells (APC) by CD4 and CD8 T cells respectively. **(B)** In indirect allorecognition, graft alloantigen (typically MHC antigen) is internalised by recipient APC [typically a dendritic cell (DC)], processed and presented as peptide fragments in the context of recipient MHC, for self-restricted recognition by recipient T cells. Although in theory both CD4 and CD8 T cells can recognise processed alloantigen via the indirect pathway, indirect pathway CD8 T cell responses are not considered relevant for the rejection of vascularized allografts. (**C)** In semi-direct allorecognition, MHC alloantigen is acquired by recipient DC but, rather than presentation as processed allopeptide, is re-presented as conformationally intact protein.

The direct pathway, whereby recipient CD4 and CD8 T cells recognize intact MHC class II and class I alloantigen, respectively on the surface of donor antigen presenting cells (APCs) (Figure 1A), was for several decades considered the dominant pathway responsible for transplant rejection. Initially proposed on the basis of the *ex vivo* mixed leukocyte reaction (4), understanding of the direct pathway has evolved, through a series of seminal publications (5–8), to encompass the passenger leucocyte theory—that allograft rejection is triggered by direct-pathway recognition of donor dendritic cells that have migrated from the allograft to host secondary lymphoid tissue.

Up to 10% of a recipient's T cells recognize a single MHC alloantigen; a peculiarity made all the more anomalous by the lack of an obvious evolutionary advantage (9–11). Two explanatory models have been proposed (12, 13): According to the high determinant density model, every MHC molecule on the surface of a donor APC is recognized as foreign, compared to only around 150 complexes per cell on host APCs following self-restricted processing and presentation of conventional antigen (14, 15). Further amplification is provided through the ability of one particular MHC alloantigen to present multiple different peptides: the multiple binary complex model. Crystallographic analysis of the interaction between an allospecific T cell and its target MHC alloantigen has revealed a similar orientation as occurs for conventional T cell responses, suggesting that the high precursor frequency of direct pathway T cell clones is principally due to multiple binary complex recognition (16, 17).

### Indirect pathway

The demonstration by Lechler and Batchelor that allografts that lacked passenger leucocytes could still be rejected (9, 10) suggested that alloantigen could also be recognized conventionally, as self-restricted processed peptide (Figure 1B). Termed the indirect pathway, its role in allograft rejection has been increasingly emphasized (11, 12, 18, 19).

Given the number of mismatched major and minor histocompatibility antigens contained within a transplanted organ, a potentially huge number of disparate allopeptide epitopes could be generated for recognition via the indirect pathway. Despite this, the alloimmune response is generally directed against a limited number of immunodominant epitopes (13–15, 20). Immunodominance is, however, not fixed and may shift with time, with patterns of dominance likely influenced by prior immunization history. Such epitope spreading may underpin chronic rejection (21).

### Semi-direct pathway

The demonstration that intact antigen could be transferred between different cell types (16, 17, 22), raised the possibility that direct pathway T cell recognition of intact alloantigen may occur on host dendritic cells (Figure 1C). This has been difficult to prove, but received experimental support from the demonstration of alloantigen transfer between cultured DCs (23), and following transfer of DCs from one mouse strain into the peritoneal cavity of another (24). Subsequent murine studies have confirmed the acquisition of intact alloantigen by recipient DCs following challenge with a vascularized allograft (25–29). The mechanisms by which alloantigen is transferred remain unclear, with early studies suggesting cell-to-cell contact was required (23, 30, 31), but more recent publications showing a role for extracellular vesicles (32, 33).

Although discussed as a distinct pathway, semi-direct allorecognition is a means by which recipient T cells may recognize “intact” alloantigen. This will result in activation of the same T cell clones as would respond via direct pathway allorecognition. In contrast, those T cell clones responding to the processed alloantigen via the indirect pathway are likely to be very different.

## The role of different allorecognition pathways in allograft rejection

The contribution of different allorecognition pathways to allograft rejection will be governed by two main factors: the presence of target epitope and the ability of that pathway, once activated, to mediate graft damage. Differences in the duration of direct and indirect T cell alloresponses are thus likely to profoundly influence their ability to mediate early and late graft rejection.

### Duration of CD4 T cell alloresponses

#### Activation of CD4 T cell clones with direct allospecificity

Experimental and human transplant studies suggest that direct pathway CD4 T cell responses are limited to the first few weeks after transplantation (34–36), with murine transplant models suggesting that its duration correlates with the lifespan of the donor DC fraction (35, 37). A small number of human studies have similarly suggested that direct pathway CD4 T cell activation is short-lived (21, 34, 38, 39).

However, recent publications from the Morelli and Benichou groups challenge these assumptions. Their studies suggest that “direct” pathway activation is largely due to recognition of intact alloantigen acquired onto the surface of host APCs by transfer of donor-derived extracellular vesicles—in essence, semi-direct allorecognition (32, 33). Whether the Morelli and Benichou findings represent a radical reappraisal of direct pathway T cell activation is not clear. Their experimental systems were not designed to examine semi-direct presentation in isolation of the conventional donor DC/ recipient T cell interaction, and it is difficult to know the relative contribution of both to T cell activation. Nevertheless, by theoretically dissociating activation of directly alloreactive CD4 T cells from expression of target MHC class II alloantigen on donor APCs, the Morelli and Benichou papers raise the potential for direct pathway CD4 T cell activation to occur at late time points after transplantation.

#### Activation of CD4 T cell clones with indirect allospecificity

It is more straightforward to theorize how indirect pathway CD4 T cell responses against self-restricted processed alloantigen can last much longer than those against intact alloantigen. In support, several animal studies have ascribed a functional role for the indirect pathway CD4 T cell response in the progression of chronic allograft rejection (40–43). Late anti-allopeptide reactivity has been similarly described in human transplant patients with chronic graft dysfunction (34, 38, 44–46), though it is unclear from these studies whether the T cell responses identified ongoing naïve responses or recall of alloreactive T cell memory established early after transplant.

On the assumption that the crux to late alloreactive T cell activation is continued presentation of stimulatory target epitope, we have recently studied the division of monoclonal populations of naive TCR-transgenic CD4 T cells that recognize a specific allopeptide epitope and that are adoptively transferred at late time points after murine heart transplantation (35). These experiments confirmed that in the mouse, direct pathway CD4 T cell activation is dependent upon the donor hematopoietic fraction, with responses not detectable beyond the first week. In contrast, chronic rejection was associated with ongoing presentation of processed MHC class I alloantigen and late activation of the responding indirect-pathway CD4 T cell population.

The indirect pathway CD4 T cell response has generally been considered as a single entity but use of monoclonal T cell lines with precise allospecificity enabled us to show that there was considerable heterogeneity within the response. Unlike the response against MHC class I allopeptide, the indirect pathway response against MHC class II allopeptide was as short-lived as the direct pathway, and not detectable beyond the first week of transplantation, because it too was dependent upon the donor hematopoietic fraction as a source of MHC class II alloantigen (35).

### Duration of CD8 T cell alloresponses

The presentation of intact MHC class I alloantigen by migrating donor DCs, in the context of pro-inflammatory co-stimulatory ligands, is generally considered the principal mechanism for generating direct pathway CD8 T cell alloimmunity. However, other than at artificially very high precursor frequencies (47), differentiation of naïve CD8 T cells to cytotoxic effectors requires help from activated allospecific CD4 T cells. Hence, the duration of the CD8 T cell alloresponse will partly be governed by availability of CD4 T cell help.

**Figure 2:**
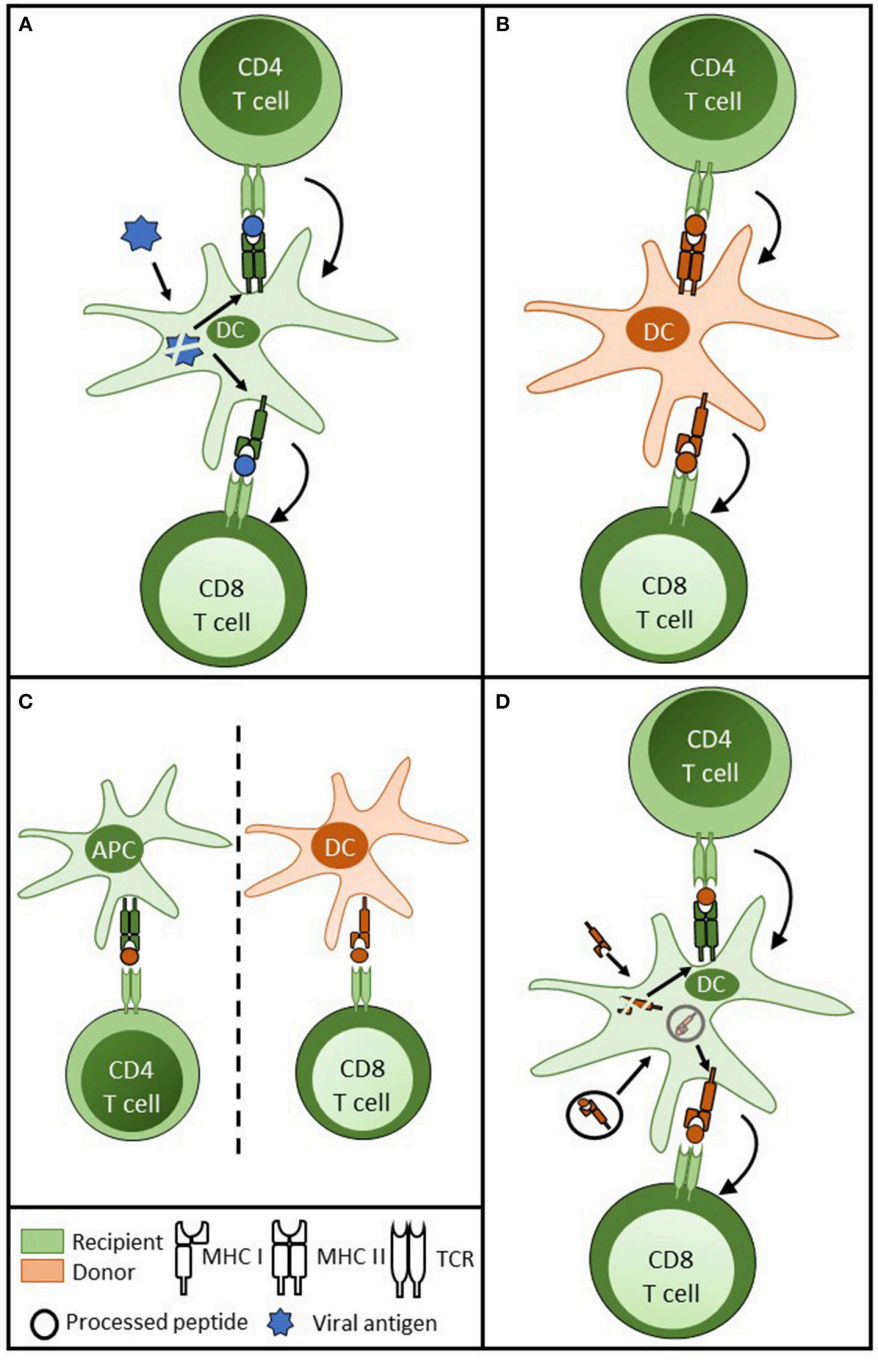
Delivery of CD4 T cell help for cytotoxic CD8 T cell alloimmunity. **(A)** For CD8 T cell responses against conventional protein antigen (such as viral antigen) following internalisation by the dendritic cell (DC), processed viral peptide is presented in the context of MHC class II and class I for CD4 and CD8 T cell recognition, respectively. CD4 T cell help is delivered to the DC, resulting in upregulation of co-stimulatory signals on the DC surface. This in turn results in enhanced presentation and more effective priming of the CD8 T cell response. **(B)** In transplantation, a similar three cell cluster model is achieved by donor DC presentation of intact MHC class II and class I to direct pathway CD4 and CD8 T cells. **(C)** Although animal models have shown that indirect pathway CD4 T cells can provide effective help for cytotoxic CD8 T cell alloresponses against intact MHC class I alloantigen, this theoretically involves a cumbersome four cell cluster with two unpaired couplets: recipient APC presenting to recipient indirect pathway CD4 T cell and donor DC presenting MHC class I alloantigen to direct pathway CD8 T cells; raising concerns regarding uncontrolled CD8 T cell alloresponses from bystander CD4 T cell activation. **(D)** These concerns are obviated if a recipient DC is able to re-present intact MHC class I alloantigen and processed MHC class I allopeptide simultaneously, enabling the provision of linked help from indirect pathway CD4 T cells to direct pathway CD8 T cells.

A series of seminal publications (48–50) have highlighted that for conventional immune responses, CD4 T cell help is delivered, not to the CD8 T cell, but through “licensing” of an intermediary APC, which, crucially, presents both MHC class I and class II restricted target epitopes for CD8 and CD4 T cell recognition, respectively. This enables the formation of a linked “three-cell” cluster (Figure 2A). With regards to alloreactive CD8 T cell alloimmunity, a similar three-cell cluster can be created that incorporates the donor DC and a direct pathway CD4 T cell (Figure 2B). Murine studies suggest this provides CD4 T cell help for generating cytotoxic CD8 T cell responses immediately after transplant (51). Thus, if help for alloreactive CD8 T cells can only be delivered by CD4 T cells with direct allospecificity, then the window for CD8 T cell activation is limited to the immediate post-transplant period.

Late CD8 T cell alloimmunity may, however, be generated through provision of help from CD4 T cells with indirect allospecificity. In support, murine studies have convincingly shown that, immediately after transplantation, indirect pathway CD4 T cells can provide CD8 T cell help (52). This raises further questions about how such help is delivered, because it requires the formation of a cumbersome four-cell cluster model (Figure 2C), in which help is delivered by CD4 T cells recognizing processed allopeptide presented by a recipient APC to CD8 T cells responding to intact MHC class I on the surface of a donor APC. Moreover, the lack of apparent linkage between the donor APC / CD8 T cell couplet and the recipient APC / CD4 T cell couplet raise concerns of inappropriate and uncontrolled CD8 T cell activation, because similar unlinked help could theoretically be provided by bystander CD4 T cell responses to unrelated antigen. Semi-direct presentation of MHC class I alloantigen by recipient DCs potentially provides an elegant solution, if one assumes that the same DCs simultaneously present class I alloantigen both as intact protein and processed peptide. This would enable formation of a three-cell cluster, in which linked help is provided by indirect pathway CD4 T cells (Figure 2D). Murine studies by ourselves and others have demonstrated simultaneous expression of intact and processed alloantigen by recipient DCs following transplantation (26, 27), and adoptive transfer studies have further suggested that these ‘cross-dressed' DCs can prime effective CD8 T cell cytotoxic alloresponses (27).

Demonstrating the functional relevance of this pathway is challenging, because it is difficult to devise model systems in which semi-direct recognition can be studied in isolation from direct pathway responses. We have shown that recipient DCs are required for generating a cytotoxic CD8 T cell population that effects rejection of heart grafts that are otherwise unable to provoke conventional direct pathway T cell responses (29). Similarly, Smyth et al. have recently demonstrated that recipients that cannot mount indirect pathway T cell responses are still capable of effecting acute cellular rejection of heart grafts, but that this rejection is dependent upon the recipient DC fraction (28). While both these papers support an independent functional role for semi-direct allorecognition in graft rejection, it should be stressed that they do not necessarily show that this pathway is dominant or more effective than conventional direct pathway responses.

In summary, animal studies provide strong support that immediately after transplantation, strong CD4 T cell-dependent cytotoxic CD8 T cell responses can be generated by direct and semi-direct presentation of class I alloantigen. The extent to which these mechanisms, particularly the semi-direct presentation of parenchymal MHC class I alloantigen, can drive late CD8 T cell activation has still to be clarified. In this respect, although clinical studies have reported late direct pathway responses in human transplant patients (53–56), this is generally based upon *in vitro* recall IFN-γ responses of recipient peripheral blood mononuclear cells (PBMCs). Definitive evidence, either experimental or clinical, for late alloreactive CD8 T cell cytotoxicity is lacking (57).

## Allorecognition pathways and effector mechanisms

### Early acute rejection

Given the above, one would anticipate that direct pathway responses dominate early after transplantation, with the CD4 T cell response central. Full CD4 T cell activation requires continued TCR engagement (58), with target MHC class II alloantigen expressed on the surface of donor APCs or re-presented by recipient DCs intact. CD4 T cells do not generally exhibit cytotoxic activity, and although direct pathway CD4 T cells can effect allograft rejection autonomously (59, 60), their greatest contribution to graft rejection is likely to be as helpers to direct pathway CD8 T cells (Figure 2B). Once activated, the cytotoxic CD8 T cell alloresponse can target all MHC class I alloantigen expressing cells of the graft.

Although indirect pathway CD8 T cell recognition of processed alloantigen can occur, this only appears relevant for the rejection of skin (61, 62) and not vascularized allografts (63), because target epitope (recipient MHC class I antigen) is expressed in the former as a consequence of host endothelial ingrowth.

Murine transplant models have confirmed that indirect pathway CD4 T cell activation also occurs early after transplantation (14, 64, 65). Although adoptive transfer studies have suggested that indirect pathway CD4 T cells may have an autonomous effector role in acute rejection (42, 66, 67), this possibly occurs only with artificially high numbers of transferred cells. As with indirect pathway CD8 T cell responses, the target allopeptide epitope for indirect pathway CD4 T cells is unlikely to be expressed at early time points within the transplant. Instead, indirect pathway CD4 T cells are uniquely capable of providing help to alloreactive B cells for generating Ig-class switched alloantibody responses (68, 69); acute alloantibody-mediated rejection is thus robust clinical evidence of early indirect pathway CD4 T cell activation.

### Late rejection

Although semi-direct presentation of endothelial MHC class II alloantigen raises the possibility of late activation of CD4 T cell clones with direct allospecificity, this remains unproven. Chronic rejection is more plausibly mediated by indirect pathway CD4 T cell responses directed against major and minor mismatched histocompatibility alloantigens (34, 38, 44–46). The principal role for indirect pathway CD4 T cells in graft rejection is likely in providing help for humoral and cytotoxic CD8 T cell alloimmunity.

As discussed above, other than development of delayed (>6 months after transplant) acute cellular rejection in human transplant recipients, definitive evidence for late allospecific cytotoxic CD8 T cell activation is lacking. One group has identified a population of circulating “terminally differentiated” effector memory (TEMRA) CD8 T cells at late time points in a cohort of kidney transplant recipients and reported a correlation with subsequent graft dysfunction (57, 70). The antigen specificity of this TEMRA population was not determined, and although it did provoke endothelial activation upon *in vitro* culture, chronic CD8 T cell stimulation in response to continued exposure to target class I alloantigen would be expected to lead to a state of exhaustion, characterized by loss of effector status (71, 72). If so, it is not immediately apparent how such exhausted cells contribute to the progression of allograft vasculopathy. Exhaustion is, however, malleable, and one possibility is that late cytotoxic CD8 T cell alloresponses are rescued from exhaustion by provision of help (73), most likely from indirect pathway CD4 T cells and formation of a three-cell cluster involving the recipient DC (Figure 2D).

In contrast, *de novo* generation of class-switched donor specific alloantibody (DSA), sometimes years after transplantation, robustly demonstrates that the indirect pathway helper CD4 T cell / allospecific B cell axis is operational at late time points after transplantation. Late-developing DSA responses are generally long-lived, suggesting deposition of allospecific long-lived plasma cells (LLPCs) in the bone marrow. These are thought to be an exclusive product of a germinal center (GC) response, and in this regard, a consistent feature in our recent murine studies on chronic heart allograft rejection is the presence of splenic GC activity at late time points after transplantation (35, 74). Long-lasting GC responses [as typically found in the gut and in humoral autoimmune disease (75, 76)] are maintained by delivery of help from specialized T follicular helper (T_FH_) cells (77), and thus their presence in our transplant models suggests ongoing T_FH_ cell differentiation from indirect pathway responses against persistently presented target allopeptide epitope. By using synthetic MHC class II / allopeptide tetramers to map the endogenous indirect pathway CD4 T cell population, we confirmed that this late presentation of allopeptide epitope was associated with ongoing division and marked late expansion (~10,000 fold) of the responding T cell population (35).

Interestingly, this expanded population also exhibited features consistent with exhaustion. Exhaustion is a state distinct from and senescence, and is characterized by progressive loss of effector function and expression of multiple inhibitory receptors (72). It has garnered much attention recently, because it is perhaps not the propensity to trigger self-reactive responses, but the ability, or otherwise, to counter their progression through development of an exhaustive state that may ultimately determine outcomes for autoimmune disease (71, 78). Our studies highlight that exhaustion is likely to impact on graft outcome, but in doing so, raises an important question: if, as seems probable, ongoing indirect pathway CD4 T cell responses against persistent allopeptide epitope are a critical factor in the progression of chronic rejection, how is this achieved despite the development of an exhausted state? This question has not been addressed experimentally, because exhaustion is only now beginning to be considered in relation to transplantation (79), but we speculate several solutions:

#### Exhausted allospecific CD4 T cells retain effector function

Exhausted T cells have been shown in some models to provide important viral control (80), which raises the possibility that exhausted indirect pathway CD4 T cells may still mediate allograft progression. Fahey et al. recently reported that in a murine model of chronic lymphocytic choriomeningitis virus infection, exhausted CD4 T cells acquire phenotypic characteristics, such as C-X-C motif chemokine receptor 5 (CXCR5), inducible T cell co-stimulator (ICOS), OX40, and PD-1, that resemble the T_FH_ subset (81). Moreover these skewed, exhausted cells expressed interleukin-21 (IL-21), a key cytokine for T_FH_ cell function (82), and could provide help for late antiviral antibody responses. Thus, these experiments may explain the simultaneous findings of exhaustion and persistent germinal center humoral immunity in our transplant model (35). Interestingly, IL-21 secretion by the helper CD4 T cell subset is also critical in preventing the development of CD8 T cell exhaustion and providing control in chronic viral infection (83–85), raising the possibility that in a transplanted individual, exhausted T_FH_-like CD4 T cells also promote the development of late cytotoxic CD8 T cell alloresponses. Not all chronic disease models, however, support a functional role for exhausted CD4 T cells in promoting late humoral immunity (86), and thus the relevance of the exhausted T_FH_ cell subset to chronic allograft vasculopathy still requires clarification. One recent study has, for example, suggested that the development of exhaustion is associated with prolonged allograft survival (87).

#### Exhaustion is bypassed by epitope diversification

One mechanism by which chronic autoimmune responses are sustained despite the propensity for exhaustion is through intra- and inter-molecular epitope diversification or “spreading” (13). As responses against a dominant epitope become exhausted, this enables the focus to shift to encompass new target T cell epitopes within the same, or completely different molecules. Diversification to sub-dominant (88), as well as cryptic self-epitopes (14, 89, 90) has been described in experimental transplant models, and a seminal publication by the Suciu-Foca group has reported an association with the development of chronic allograft vasculopathy in human heart transplant recipients (21). Epitope spreading, even to target a second alloantigen on the graft, may permit humoral responses against the first alloantigen to be maintained, because of the ability of the allospecific B cell to acquire additional graft alloantigen as it internalizes target alloantigen (91).

#### Recall of memory CD4 T cell responses

In contrast to murine transplant models, which generally study naïve T cell responses to alloantigen, recall memory responses, often from cross-reactive heterologous immunity, are considered a sizeable component of the alloresponse encountered in clinical practice (92–94), and are particularly relevant because of their relative insensitivity to immunosuppressive agents. Of note, unlike central memory alloreactive CD4 T cells (generated following acute allograft rejection), exhausted alloreactive CD4 T cells (purified from recipients undergoing chronic rejection) were unable to provide co-stimulation-independent help for the production of alloantibody in our experimental system (35). This raises the possibility that in clinical practice, T cell help for generating late humoral alloimmunity is provided by recall responses of allospecific memory CD4 T cells that have been deposited early after transplantation, rather than by a chronically activated, but exhausted, population. Certainly, it is unlikely that exhausted CD4 T cells will respond to target epitope in the typical ELISpot assays used clinically to evaluate late direct and indirect pathway activation (53–56); these assays are instead generally considered a marker of memory recall, particularly those that involve prolonged *in vitro* culture (21). This may explain why in clinical transplantation late *de novo* donor specific alloantibody responses appear to focus on disparate MHC class II, rather than MHC class I, alloantigens (95, 96). Although MHC class II alloantigen is likely to be upregulated on the endothelium of human allografts, MHC class I alloantigen will still be expressed more widely and much more abundantly within the graft, and certainly there is no *a priori* reason why disparate MHC class I alloantigens will be less immunogenic than disparate class II alloantigens. Thus, MHC class I alloantigen may be presented as processed allopeptide continually, resulting in chronic activation and exhaustion within the responding helper CD4 T cell population, whereas levels of MHC class II alloantigen expression within the graft may fluctuate and fall below a threshold at which the CD4 T cell response terminates and effective anamnestic responses are generated. Subsequent upregulation of class II alloantigen expression on the graft endothelium in response to stress from, for example, concurrent viral infection, may result in provision of help for generating late anti-class II alloantibody through recall responses of deposited memory class II allopeptide-specific CD4 T cells. Although speculative, this would be consistent with the reported association between the development of class II allopeptide-specific CD4 T cell memory responses (as determined by *in vitro* ELISpot culture assay) and chronic rejection in human heart transplant recipients (21).

## Targeting late T cell alloresponses to prevent progression of chronic rejection

A better understanding of the allorecognition pathways active at late time points after transplantation will inform the development of tolerogenic strategies that aim to prevent progression of allograft vasculopathy and prolong allograft survival. Two approaches will be considered further: regulatory T cell therapy and targeting the metabolic pathways that sustain chronic T cell alloresponses.

### Regulatory T cells

Numerous cells with immunoregulatory potential are described (97). Here we focus on the classical CD25^pos^ CD4 T regulatory cell (T-reg) (98, 99). Naturally-occurring, thymus derived T-regs (nT-regs) are defined by the master transcription factor forkhead box P3 (FOXP3) (100, 101) and provide essential control of autoimmunity through: absorption of pro-inflammatory IL-2; CTLA-4-mediated masking of CD80 and CD86 co-stimulatory ligands on APCs; expression of immune-inhibitory molecules (IL-10, IL-35, TGF-β); and granzyme-mediated killing of APCs (102).

FOXP3-expressing T-regs develop in the periphery (pT-reg) upon engagement with target epitope in a TGF-β rich environment (103, 104). While nT-regs are polyclonal, pT-regs are defined by antigen exposure. This has important consequences for T-reg therapy, because although T-regs may exhibit non-antigen-specific suppressor function, more potent inhibition occurs upon engagement of the TCR. In support, pre-clinical studies suggest that alloantigen-specific induced T-regs (iT-reg) are more effective than nT-regs in preventing allograft rejection (105–107).

Current translational transplant studies are focused upon the delivery of T-regs with direct allospecificity (97, 99, 108–112), partly because these can be generated more readily than indirect pathway T-regs. These are likely to be most effective in preventing early acute rejection, albeit the strong pro-inflammatory environment immediately after transplantation may favor deleterious trans-differentiation of the administered T-regs to T effector status (113). Control of acute rejection is not a major clinical problem, and while early administration of direct pathway T-regs may have long-lasting consequences (114), based on the above consideration of expression of target epitope at late time points, one would predict that chronic rejection will be better controlled by iT-regs with indirect allospecificity. This is supported by murine studies (106, 107, 115, 116). If so, the challenge will be in determining not only which mismatched major and minor histocompatibility alloantigens are being actively processed, but also the precise self-restricted allopeptide epitopes that are generated by this processing. This may not be as daunting as first appears, because as long as target epitope is expressed, transfer of allopeptide-specific T-regs may dominantly inhibit concurrent indirect pathway responses against other alloantigens (117). Subsequent epitope diversification that shifts the focus of the indirect pathway CD4 T cell response to new epitopes on different alloantigens should be similarly controlled by “infectious” tolerogenic mechanisms (118, 119).

### T cell metabolic pathways

It is likely that the alloreactive CD4 T cell response at late time points after transplantation will comprise a number of different populations with specificities for different alloantigens. These populations will further differ in their helper T cell subset polarization (T_H_1, T_H_2, T_H_17, T_FH_), and in their stage of effector to memory transition. This will include populations of allospecific CD4 T cells that have acquired an exhausted phenotype or have undergone peripheral differentiation into regulatory T cells (pT-reg). As discussed above, the relative contribution of these different populations to the progression of allograft vasculopathy has still to be clarified, but it is now apparent that the individual stages of T cell differentiation are underpinned by profoundly different metabolic states, and it is the metabolic environment that dictates T cell differentiation (120–122). Immunometabolism is still an emerging field but raises the potential that specific metabolic pathways could be targeted with the expectation of improvements in transplant outcomes.

**Figure 3:**
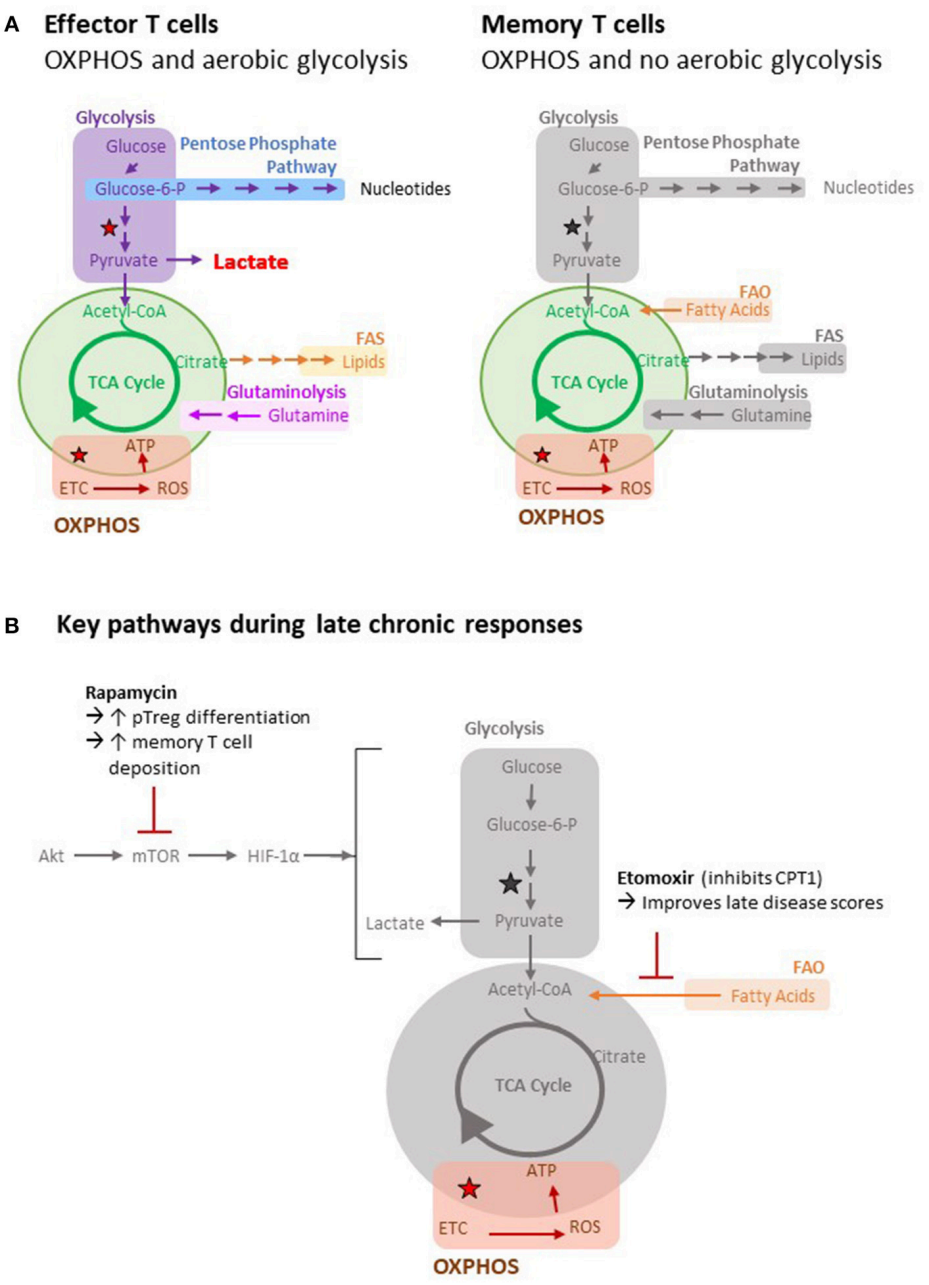
Metabolic pathways of T cells. **(A)** Important metabolic pathways in T cells include glycolysis, the tricarboxylic acid (TCA) cycle (green), fatty acid oxidation (FAO), fatty acid synthesis (FAS), oxidative phosphorylation (OXPHOS) and glutaminolysis. The stars indicate reactions that generate reducing equivalents (i.e. NADH) to drive OXPHOS. Utilization of different metabolic pathways by different cell types is indicated. Greyed out pathways represent pathways that have not been defined. **(B)** Key metabolic pathways and inhibitors during late chronic responses are highlighted pathways by different cell types is indicated.

Naïve T cells persist in a catabolic state, with their bioenergetic requirements met largely by mitochondrial oxidative phosphorylation (OXPHOS). Upon binding target antigen, activated T cells switch their metabolic profile to aerobic glycolysis, characterized by marked augmentation in glycolysis and a lesser, but nevertheless critical (123), increase in OXPHOS (Figure 3). Central to these changes are signaling via the phosphoinositide 3-kinase (PI3K)–AKT1–mammalian target of rapamycin (mTOR) axis (124–126) and upregulation of the transcription factors MYC and hypoxia-inducible factor 1α (HIF1α). This results in increased amino acid and glutamine transfer to fuel glutaminolysis and glycolysis. Although the switch to glycolysis is clearly, when oxygen is otherwise abundant, an inefficient means of producing ATP; it does generate the metabolic intermediates for synthesizing the nucleotides and amino acids required for differentiation and division. It also produces acetyl-CoA for manufacturing lipids (127). Upon pathogen clearance, the activated T cell population undergoes contraction by apoptosis, leaving a small population of long-lived memory T cells. These cells revert to a catabolic state, but unlike naïve cells, OXPHOS is maintained, at least in part, by mitochondrial fatty acid oxidation (FAO), in which IL-7 and IL-15 signaling mediates AMP-activated protein kinase (AMPK)-dependent increases in mitochondrial biomass and spare respiratory capacity (128–131).

Not all effector T cell populations rely upon aerobic glycolysis, and most notably, pT-regs rely upon OXPHOS and FAO metabolism (132, 133). Consequently, inhibition of glycolysis, either by blocking mTOR1c or downstream HIF1 α signaling, promotes a switch from T_H_17 to pT-reg differentiation (134–136). The T_FH_ subset is similarly more dependent upon OXPHOS than the classical T_H_1 subset (137), and Bcl-6 expression (the key transcription factor for this subset) represses glycolysis (138). This possibly counters the high glucose requirements associated with the germinal center B cell response. Finally, although exhausted T cells exhibit typical aerobic glycolysis at initiation of the response, continued antigen binding to the TCR results in downregulation of the PI3K–AKT1–mTOR signaling pathway (139) and NFATC-mediated expression of PD-1 and other inhibitory ligands (140, 141). This results in inhibition of glycolysis and increased FAO (142).

From the discussions presented so far, we make two predictions: firstly, that at late time points graft alloantigen will be continually processed by recipient APCs for recognition by CD4 T cells with indirect allospecificity; and secondly, that the metabolic profile governing these late chronic responses will be skewed from glycolysis and instead focus on OXPHOS, perhaps with a reliance on FAO. If so, then transplant outcomes will be determined by the relative contributions of the indirect pathway T cell subsets that meet those energy requirements, with memory or T follicular helper cell responses being potentially destructive, while exhausted or regulatory T cell responses being neutral or beneficial. Therapeutic targeting of immunometabolic pathways is a developing field, and approaches so far in autoimmunity (143) and transplantation (144) have generally focused on inhibiting glycolysis and preventing the differentiation of T effector cells. Such an approach may not be particularly effective if commenced at late time points after transplantation when glycolysis is not the dominant metabolic pathway. In this regard, Byersdorfer et al. have recently shown that the administration of etomoxir, an agent that blocks FAO by irreversibly inhibiting carnitine palmitoyl-transferase 1 (CPT1), improved late disease scores in a murine model of chronic graft-vs.-host disease (145). Interestingly, etomoxir only affected donor alloreactive T cells that had divided more than six times and was ineffective at preventing acute T effector differentiation, suggesting that similar inhibition of FAO may prevent progression of allograft vasculopathy in recipients of solid organ transplants. One potential problem with this approach is that the metabolic pathways active in the different subsets of exhausted, memory, pT-reg, and T_FH_ cells may be quite similar. For example, while treatment with the mTOR inhibitor rapamycin would, by blocking glycolysis, be expected to promote the development of pT-regs and favor allograft survival (134, 136, 146), it has also been shown to increase deposition of memory T cells (130, 147); a population that, as discussed above, may have a contrary impact on graft outcomes. The same considerations hold for other agents, such as 2-deoxyglucose, that potentially increase pT-reg generation by blocking glycolysis (144, 148).

As the field of immunometabolism advances, it is likely that more nuanced differences in the metabolic profiles of memory, regulatory and follicular helper CD4 T cells will become apparent, and that these differences could ultimately be targeted pharmacologically. For example, the phosphatase PTEN, which is the main negative regulator of PI3K, has been recently shown to be critical for maintaining T-reg stability (149, 150). Its conditional deletion led to increased glycolysis and re-differentiation of the T-reg population into other T helper cell subsets, most notably the T_FH_ cell subset, and was associated with development of germinal center humoral autoimmunity (150).

## Summary

Although the CD4 T cell alloresponse is a key determinant of transplant outcomes, many aspects of this response remain unclear more than 60 years after the first human kidney transplant. It seems likely that the late CD4 T cell response will be focused on self-restricted, processed alloantigen. This response will be fluid and shift to target different epitopes on the same or different alloantigens and involve different T helper cell subsets at varying stages of effector and memory differentiation. Although the future introduction of new broad acting immunosuppressive agents is likely to improve transplant outcomes, their impact may only be modest (151). Instead, alloantigen-specific cellular therapies, such as the administration of cultured T-regs, are on the cusp of entering clinical practice, and potentially offer a personalized approach to specifically inhibit deleterious alloimmune responses that are active in a particular recipient, while preserving global immune responsiveness. Key to the success of these cellular therapies will be the ability to interrogate accurately the entire CD4 T cell alloresponse in the individual, and to map currently active effector or exhausted CD4 T cell responses, as well as the recent deposition of central and effector memory T cells. This will require the development of experimental approaches that analyze the allospecific T cell population *in situ*, without the need for protracted *in vitro* culture and stimulation.

## Author contributions

All authors contributed equally to the design and preparation of this review. JS and GP conceptualized and designed the figures.

### Conflict of interest statement

The authors declare that the research was conducted in the absence of any commercial or financial relationships that could be construed as a potential conflict of interest.
